# Diagenetic and shear-induced transitions of frictional strength of carbon-bearing faults and their implications for earthquake rupture dynamics in subduction zones

**DOI:** 10.1038/s41598-019-44307-y

**Published:** 2019-05-27

**Authors:** Shunya Kaneki, Tetsuro Hirono

**Affiliations:** 1 Department of Earth and Space Science, Graduate School of Science, Osaka University, Toyonaka, Osaka, 560-0043 Japan; 2 Present Address: Disaster Prevention Research Institute, Kyoto University, Uji, Kyoto, 611-0011 Japan

**Keywords:** Geology, Geophysics

## Abstract

Subduction-related diagenetic reactions affect fault strength and are thus important for understanding earthquake rupture dynamics in subduction zones. Carbonaceous material (CM) is found worldwide in active plate-boundary and intracontinental faults, yet the effect of its transformation on frictional strength and rupture dynamics remains unknown. We conducted high-velocity friction experiments together with organochemical analyses on CM in the form of lignite, bituminous coal, anthracite and graphite. Results clearly show that an increase in CM maturity and crystallinity leads to a decrease in the peak friction coefficient (from 0.5 to 0.2). We also infer that friction applied to low-grade CM increases its maturity, but friction applied to high-grade CM reduces its maturity. These findings suggest that both diagenetic and shear-induced transformations of CM strongly affect the frictional strength of CM-bearing faults, potentially affecting the depth-dependences of frictional strength and rupture dynamics on plate-subduction faults.

## Introduction

Earthquakes at and around plate-subduction zones sometimes generate gigantic tsunamis that endanger coastal areas. Understanding the effect of diagenetic reactions that change the physical, hydrological and thermal conditions and processes at plate interfaces is important for understanding earthquake dynamics in subduction zones^1,2^. The links between diagenesis and earthquake dynamics along the shallow parts of plate subduction interfaces (depths of up to several tens of kilometres) have been attributed to changes of the chemistry of fault-forming materials, changes of local physical and hydrological properties and changes of pore-fluid pressures^3^, all of which are related to smectite to illite transformations^4–7^, as well as crystallization of opal to quartz^5,8^, crystallization of palagonite to smectite^8^ and lithification processes^1,2,7,8^. For example, it is likely that large historical earthquakes (*M*_w_ ≥ 6.9) offshore Costa Rica were associated with dehydration of smectite and illitization-induced silica cementation^7^. Similarly, the 2004 Sumatra-Andaman earthquake (*M*_w_ = 9.2) was driven by dehydration of silicates before subduction^8^. However, the relationship of diagenetic thermal maturation of carbonaceous material^9^ to fault strength and earthquake rupture dynamics remains poorly understood.

The frictional strength of faults within the brittle crust is an important controlling factor in earthquake mechanics; it governs the dynamics of earthquake nucleation and rupture propagation^10^. The results of conventional geological and seismological observations and laboratory friction experiments suggest that some faults within the brittle crust are frictionally weak^11–15^. Plausible explanations for fault weakness include the presence of frictionally weak materials^13–15^, high pore-fluid pressures^11,12^, foliation fabrics in fault rocks^16^ and dynamic fault-weakening processes^17^. In particular, the abundance of weak clay minerals has been widely used as an explanation for fault weakness in subduction zones^18^.

**Figure 1:**
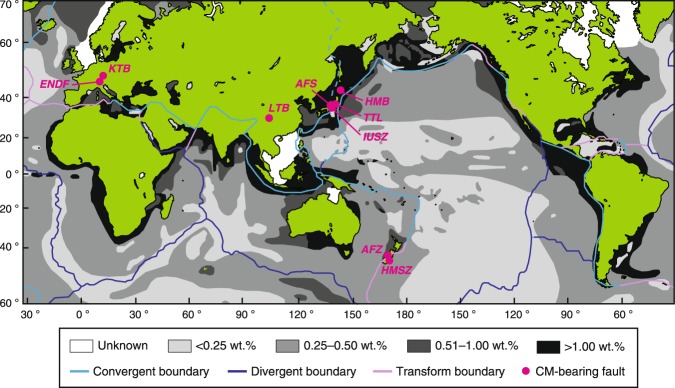
Worldwide distribution of carbon-bearing faults. Map showing concentrations of CM in oceanic sediments^29^ and major plate boundaries. *ENDF*, Err Nappe detachment fault^26^; *KTB*, KTB borehole^28^; *LTB*, Longmenshan thrust belt^24^; *AFS*, Atotsugawa fault system^14^; *IUSZ*, Inuyama-Unuma shear zone^23^; *TTL*, Tanakura Tectonic Line^27^; *HMB*, Hidaka metamorphic belt^25^; *AFZ*, Alpine fault zone^20^; *HMSZ*, Hyde–Macraes shear zone^22^.

Carbonaceous material (CM) is another common material in the brittle crust. Carbon-bearing rocks are common in both plate-boundary faults^19–21^ and intracontinental active faults^14,22–28^, and in seafloor sediments near plate-subduction trenches^29^ (Fig. 1). Laboratory experiments have shown that CM has lower frictional resistance^27,30–33^ than those of crust-forming rocks on Earth such as granite and sandstone^34^, and its presence weakens fault strength and affects rupture process^24,31,35–38^. Furthermore, the increase of the thermal maturity of CM as a result of diagenetic reactions^9^ probably affect its frictional properties^27,39^. However, we know little about the relationship between CM transformation (maturation of CM) and frictional properties in carbon-bearing faults.

To examine this relationship, we performed chemical analyses, X-ray diffraction measurements and transmission electron microscope (TEM) observations, and infrared (IR) and Raman spectroscopic analyses on four non-deformed samples of simulated carbon-rich fault material (lignite, bituminous coal, anthracite and graphite) and then, using those samples, conducted rotary-shear friction experiments designed to reproduce seismic slip. We then carried out TEM and scanning electron microscope (SEM) observations and IR and Raman spectroscopic analyses on the CM samples after the friction experiments. Descriptions of the CM samples we used and the details of the analytical techniques we employed are provided in the Methods section.

On the basis of our analytical results, we examined the relationships between the frictional properties of the stimulated fault materials and their organochemical characteristics and considered the possible implications of these for the depth-dependent frictional strength of carbon-bearing faults and their role in rupture dynamics in subduction zones.

## Results

### Chemistry of initial (non-deformed) CM samples

Elemental analyses showed the atomic O/C and H/C molar ratios of the initial samples to be respectively 0.37 ± 0.03 and 1.93 ± 0.11 for lignite, 0.07 ± 0.00 and 1.58 ± 0.16 for bituminous coal, 0.03 ± 0.00 and 0.74 ± 0.08 for anthracite and 0.00 ± 0.00 and 0.00 ± 0.00 for graphite (Supplementary Table S1).

**Figure 2:**
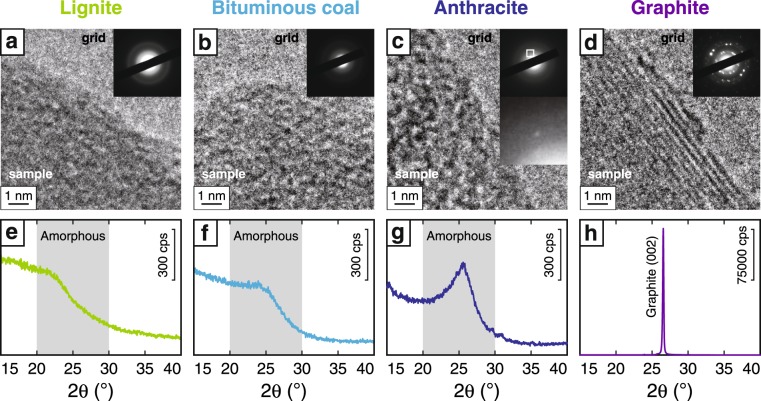
Crystallinity of non-deformed CM samples. Results of TEM observations of CM before friction experiments showing nanoscale structure and diffraction patterns (**a**–**d**) and XRD profiles of CM (**e**–**h**).

Transmission electron microscope (TEM) observations of the lignite and bituminous coal samples showed an absence of crystalline structure without distinct diffraction patterns (Fig. 2a,b), whereas the anthracite showed a small diffraction pattern (Fig. 2c) and the graphite showed well-ordered crystalline structure with a clear diffraction pattern (Fig. 2d). X-ray diffraction (XRD) profiles of the lignite and bituminous coal samples showed no distinct peaks, but each showed a broad peak at 2θ = 20°–30° indicating an amorphous structure (Fig. 2e,f). In contrast, the XRD profile of the anthracite sample showed a relatively sharp peak at around 2θ = 25.4° (Fig. 2g) and that of graphite showed a strong peak at 2θ = 26.7° (Fig. 2h).

### Frictional properties of CM

We conducted rotary-shear friction experiments on the CM samples at normal stresses of 0.5, 1.0, 1.5 and 2.0 MPa, slip rate of 1 m s^−1^ and slip distance of 15 m under water-saturated conditions at room temperature. We used a rotary-shear friction apparatus (Supplementary Fig. S1) to obtain the changes of shear stress of CM with displacement. For each experiment, two cylinders of Berea sandstone were first immersed in distilled water under vacuum for 1 h. CM powder (particle size ≤75 µm) was then placed on the circular surface of the sandstone cylinder to be used on the rotating side of the friction apparatus and saturated with 1.0 mL of distilled water. The two cylinders were then placed end to end with the CM between them (Supplementary Fig. S1) and enclosed in a polytetrafluoroethylene sleeve. The sample assemble was again immersed in distilled water under vacuum for 1 h. After placing the sample in the apparatus, it was rotated 2–3 times under normal stress of ~0.4 MPa to ensure a uniform thickness of gouge and then, after application of the target normal stress, it was held stationary for 30 min. For all experiments, the thickness of the CM after pre-compaction was about 1.3 mm. During pre-compaction, the sample was wrapped with a water-saturated cleaning tissue and polyolefin film to prevent drying of the CM. Because the presence of oxygen can strongly affect transformation reactions of CM^40^ and the natural condition of faults at depth is expected to be anoxic^41^, before each experiment, N_2_ gas was flowed through the apparatus for 5 min at a pressure of 0.1 MPa to prevent oxidation of CM during the experiments. The raw torque data were acquired at a frequency of 200 Hz. Torque data acquired before each experiment were used for background correction of the raw data, and then the torque data between the Berea sandstone cylinders and the polytetrafluoroethylene sleeve was subtracted (Supplementary Fig. S2). After each experiment, the sample assembly was dried at 50 °C for several hours. The outer 6.0–12.5 mm of sheared CM gouge was then collected for spectroscopic analysis.

We applied the following empirical exponential slip-weakening friction law to determine the best-fit curves for the changes of shear stress with displacement:

1 $$\tau ={\tau }_{{\rm{d}}}-({\tau }_{{\rm{d}}}-{\tau }_{{\rm{p}}})\,\exp \,(-\frac{D}{{D}_{{\rm{c}}}})$$

where *τ* is shear stress (MPa) as a function of displacement, *D* is displacement (m), *τ*_d_ is dynamic shear stress (MPa), *τ*_p_ is peak shear stress (MPa) and *D*_c_ is critical slip-weakening distance (m). We then applied the empirical law to determine best-fit curves for the calculated values of *τ*_p_, *τ*_d_ and *D*_c_:

2 $${\tau }_{{\rm{p}}({\rm{d}})}={\mu }_{{\rm{p}}({\rm{d}})}{\sigma }_{{\rm{n}}}+C$$

3 $${D}_{{\rm{c}}}=\alpha {{\sigma }_{{\rm{n}}}}^{-\beta }$$

where *μ*_p_ and *μ*_d_ are peak and dynamic friction coefficients, respectively, *σ*_n_ is normal stress (MPa), *C* is a cohesion term (MPa) and *α* and *β* are experimentally determined coefficients.

**Figure 3:**
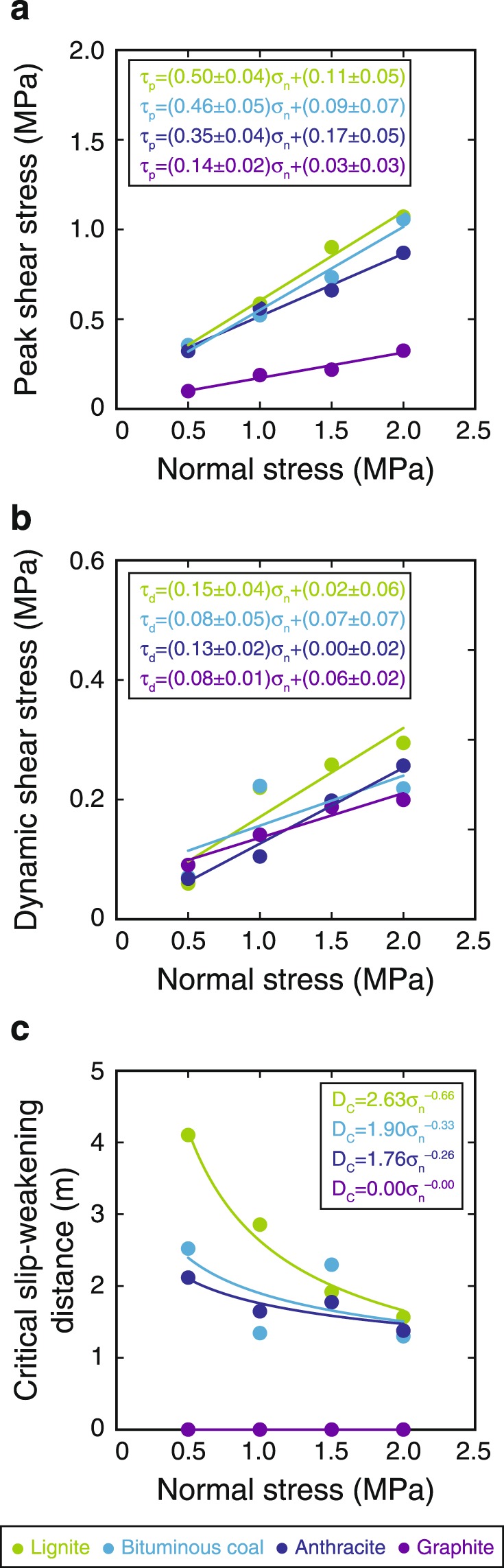
Determination of frictional properties of CM samples. Estimated values of peak shear stress (*τ*_p_) (**a**), dynamic shear stress (*τ*_d_) (**b**) and critical slip-weakening distance (*D*_c_) (**c**) plotted against normal stress. Fitting errors and intercepts of best-fit slopes for peak and dynamic friction are also shown.

All of the samples showed clear decreases in shear stress with increasing slip distance under all normal stress conditions (Supplementary Fig. S3), and the data were well fitted by Equation (1). The estimated values of *τ*_p_ and *τ*_d_ show strong linearity but the slopes of the best-fit lines differ according to the type of CM tested (Fig. 3a,b), except for *τ*_d_ of bituminous coal. Although the *μ*_p_ values for lignite and bituminous coal were almost the same (~0.5), those of anthracite and graphite were slightly and markedly lower (0.3–0.4 and 0.1–0.2, respectively) (Fig. 3a). All of the samples showed similar *μ*_d_ values of ~0.1–0.2 (Fig. 3b). *D*_c_ values decreased with increasing *σ*_n_ and were well fitted by Equation (3) for all samples except graphite, for which *D*_c_ values were zero for all stress conditions (Fig. 3c). Although the calculated values of *α* and *β* differed slightly (0.00 to 2.63 and 0.00 to 0.66, respectively) depending on the type of CM tested, the values obtained are negligible compared to those of other rock-forming minerals^17^ (3–78 and 1.13–1.24 for *α* and *β*, respectively).

The SEM images we obtained for all four types of CM showed homogeneous structures without shear fabrics such as R1 Riedel or Y shear planes (Supplementary Fig. S4), thus indicating no correlation between their frictional properties and their microstructures.

### Changes in organochemical characteristics of CM after friction experiments

**Figure 4:**
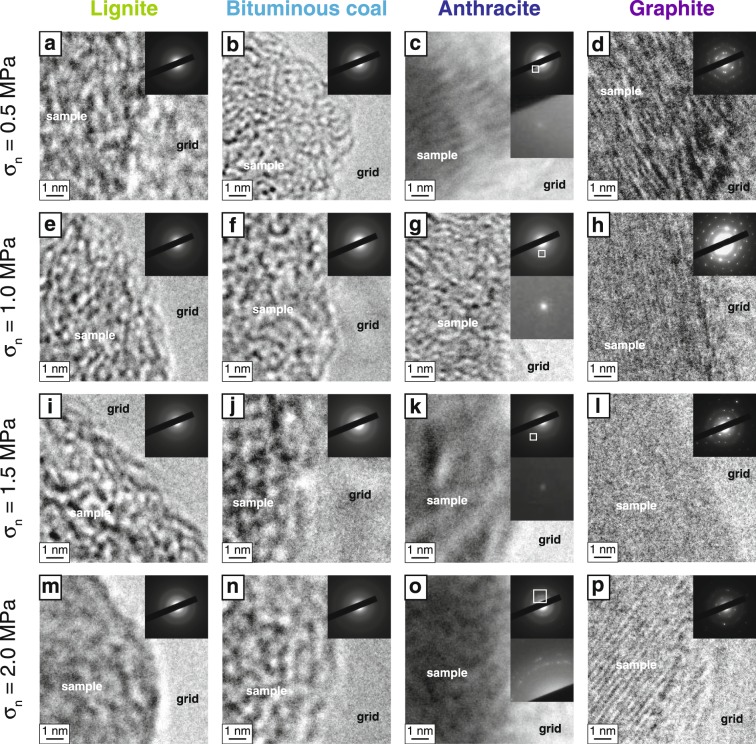
Crystallinity of CM samples after friction experiments. Results of TEM observations of four types of CM after friction experiments showing nanoscale structure and diffraction patterns at normal stresses of 0.5, 1.0, 1.5 and 2.0 MPa.

We applied TEM microscopy and IR and Raman spectroscopy to the CM samples before and after each friction experiment. TEM observations of the lignite and bituminous coal samples after friction experiments under all stress conditions showed no crystalline structure and no distinct diffraction patterns (Fig. 4a,b,e,f,i,j,m,n), similar to the TEM observations of the non-deformed lignite and bituminous coal samples (Fig. 2a,b). Although the anthracite sample showed a small diffraction pattern after friction experiments at normal stresses of 0.5, 1.0 and 1.5 MPa (Fig. 4c,g,k), similar to the non-deformed anthracite (Fig. 2c), a clear diffraction pattern appeared after the friction experiments at normal stress of 2.0 MPa (Fig. 4o). The graphite sample showed a well-ordered crystalline structure with a clear diffraction pattern after friction experiments at 0.5 and 1.0 MPa (Fig. 4d,h), but a relatively weak diffraction pattern was observed after experiments at 1.5 and 2.0 MPa (Fig. 4l,p).

**Figure 5:**
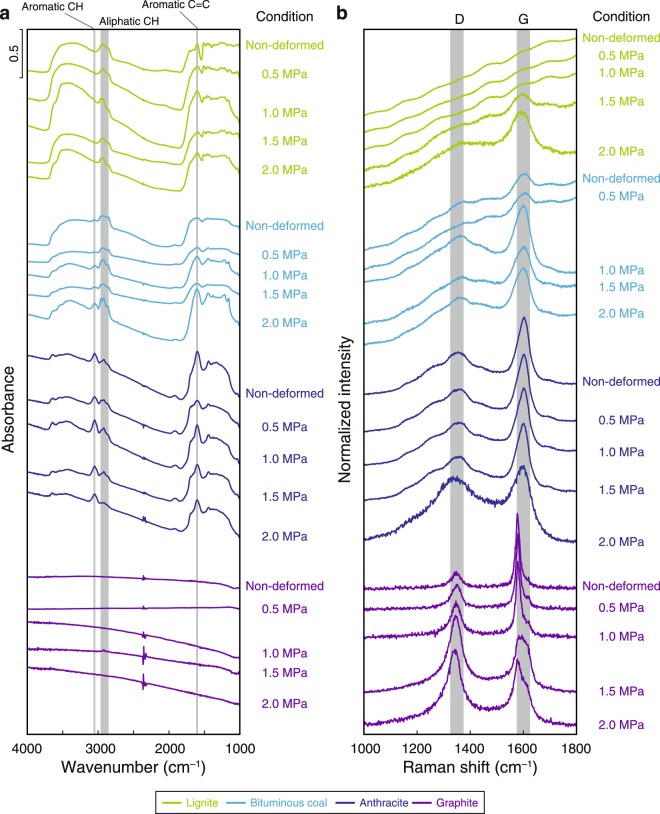
Results of spectroscopic analyses of CM samples. Representative IR spectra (**a**) and Raman spectra (**b**) of CM before and after friction experiments at normal stresses of 0.5, 1.0, 1.5 and 2.0 MPa.

The IR spectra of CM characteristically show various absorbance peaks that correspond to organic chemical bonds^42^. In this study, we focused on an aromatic C–H band (3050 cm^−1^), aliphatic C–H bands (2960, 2930 and 2860 cm^−1^) and an aromatic ring C=C band (1600 cm^−1^) (Fig. 5a).

Lignite samples showed sharp absorbance peaks of aliphatic C–H and aromatic C=C bands before and after all friction experiments. Bituminous coal samples also showed absorbance peaks of aliphatic C–H and aromatic C=C bands before and after experiments at 0.5 MPa, and aromatic C–H bands appeared in the IR spectra of these samples after experiments at 1.0, 1.5 and 2.0 MPa. Anthracite samples showed absorbance peaks of aromatic C–H, aliphatic C–H and aromatic C=C bands before and after friction experiments at 0.5, 1.0 and 1.5 MPa, whereas those of aliphatic C–H bands almost disappeared after experiments at 2.0 MPa. Graphite samples showed no significant absorbance peaks in IR spectra before and after all friction experiments.

Raman spectra of CM characteristically show strong peaks at around 1360 and 1600 cm^−1^, which are referred to as disordered (D) and graphite (G) bands, respectively^43^. Representative Raman spectra of each sample are shown in  Figure 5b. To compare the spectral features of the Raman spectra, the spectra were normalized to have the same height.

Raman spectra of lignite samples before and after friction experiments at 0.5 and 1.0 MPa showed no clear D- and G-band peaks. After experiments at 1.5 MPa they showed a peak of the G band, and after 2.0 MPa they showed significant peaks of both D and G bands. Bituminous coal samples before and after friction experiments at 0.5 MPa showed small D- and G-band peaks and the intensities of both bands strengthened further after experiments at 1.0, 1.5 and 2.0 MPa. Anthracite samples showed clear D- and G-band peaks before and after all experiments; after experiments at 2.0 MPa the peak of the D band was the strongest we observed among the anthracite samples before and after friction experiments. Graphite samples before the experiments showed a small D-band peak and strong G-band peak and the intensity of D band increased with increasing normal stress.

**Figure 6:**
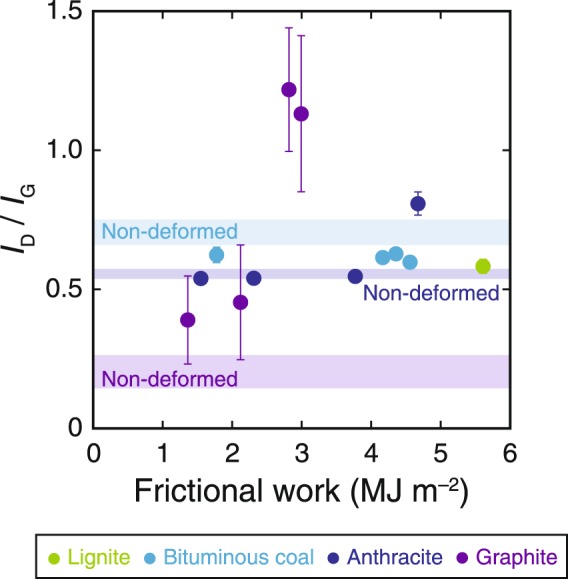
Relationship between Raman *I*_D_/*I*_G_ ratios and frictional work. The method used for calculation of the *I*_D_/*I*_G_ ratio is described in the Methods section. Error bars indicate plus or minus one standard deviation (*n* = 30).

To further quantify the relationship of the Raman spectra to the frictional properties of CM, we plotted the ratio of the intensities of the D and G bands (*I*_D_/*I*_G_) versus frictional work, defined as the integral of the shear strength over the local slip (Fig. 6). For lignite samples, we calculated *I*_D_/*I*_G_ only for experiments at 2.0 MPa because no strong D band was observed under lower pressure conditions.

The *I*_D_/*I*_G_ ratio of bituminous coal samples decreased slightly after all friction experiments. For anthracite samples, *I*_D_/*I*_G_ changed little until the experiment at 2.0 MPa, when *I*_D_/*I*_G_ increased considerably. For graphite samples, *I*_D_/*I*_G_ showed a quasi-systematic increase with increasing frictional work.

## Discussion

Our analyses revealed differences in the elemental compositions of the four types of non-deformed CM (Supplementary Table S1). The O/C ratio of the lignite sample was considerably higher than those of the bituminous coal, anthracite and graphite samples, all of which were of similar magnitude. H/C ratios decreased systematically with increasing CM maturity (from lignite to graphite). These changes are consistent with the results of previous studies that have reported changes in elemental compositions related to diagenetic increases of the maturity of CM^44,45^, and suggest that the four types of CM we used are excellent analogues for CM at different depths and temperatures in natural plate-subduction zones.

TEM observations and XRD measurements clarified the differences in the crystallinity of the four types of non-deformed CM we used. The lignite and bituminous coal samples were completely amorphous, with no diffraction patterns in TEM images (Fig. 2a,b) and a clear broad peak at 2θ = 20°–30° in XRD profiles (Fig. 2e,f). Anthracite showed a weak diffraction pattern in a TEM image and a relatively sharp peak at 2θ = 25.4° in an XRD profile (Fig. 2c,g), strongly suggesting a partly crystallized structure. Graphite was fully crystallized with a clear diffraction pattern and a very sharp peak at 2θ = 26.7° (Fig. 2d,h).

The values of the frictional shear strength of the CM samples peaked at the initial stage of each friction experiment and then decreased dynamically with increasing slip (Supplementary Fig. S3). In the past, the initial *τ*_p_ for graphite has been considered to represent the work required to rotate particles so that their weak (001) planes are subparallel to the shear direction^46^. We now consider several physicochemical processes that have been suggested as possible contributors to the dynamic weakening processes, including frictional melting^47^, silica gel formation^48^, flash heating^49^ and thermal pressurization^50^.

Given that frictional work is mostly converted to heat during frictional slip^51^, the lignite samples after experiments at 2.0 MPa were subjected to the greatest amount of work (Fig. 6) and, hence, to the highest temperatures. The IR spectra of lignite samples before and after the friction experiments showed clear absorbance peaks of aliphatic C–H and aromatic C=C bonds, which have been reported to disappear at ≥700 and ≥800 °C, respectively^52^, thus indicating that the maximum temperature during friction experiments should not exceed 700 °C. Because the melting point of CM is several thousands of degrees Celsius^53^, we can exclude the possibility of frictional melting of CM contributing to dynamic weakening. CM samples were treated with acids in order to remove silica, so we can also exclude the possibility of silica gel formation. Previous friction experiments using two types of host rock^15^ have reported that because of the high permeability of Berea sandstone, its use in friction experiments should prevent thermal pressurization. Because we used Berea sandstone as the host rock in the friction experiments, we can thus exclude thermal pressurization as a contributor to dynamic weakening. On the basis of the above exclusions, we therefore infer that flash heating may be the dominant process that caused dynamic fault weakening during friction experiments.

**Figure 7:**
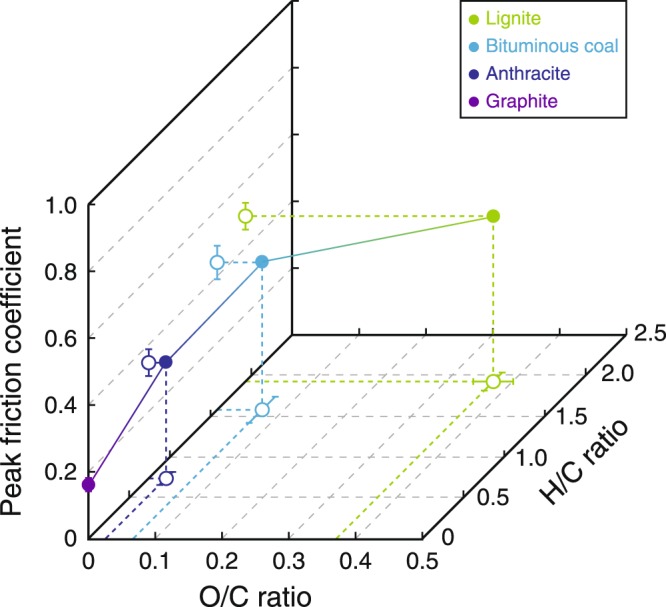
Relationships between peak friction and H/C and O/C ratios of CM samples. Errors of peak friction and atomic ratios were derived from fitting errors and standard deviations (*n* = 5), respectively (Supplementary Tables S1–5).

Previous high-velocity friction experiments on amorphous carbon samples similar to the lignite and bituminous coal used in this study revealed *µ*_p_ and *µ*_d_ values of 0.54 and 0.15, respectively^27^; these values are well consistent with our experimental results (Fig. 3a,b). Natural CM-bearing fault rocks or their surrounding host rocks, such as those of the Longmenshan fault that slipped during the 2008 Wenchuan earthquake, include ~25–30 wt.% poorly crystallized CM and have *µ*_p_ and *µ*_d_ values of 0.47–0.53 and 0.13–0.16, respectively^24,35^; these characteristics are also well consistent with our results (Fig. 3a,b). Among the frictional properties we determined (Fig. 3), only the peak friction coefficient *μ*_p_ showed a systematic change related to the chemistry of CM, which means that it is a crucial factor when considering friction and rupture dynamics along carbon-bearing faults. Results showed that *μ*_p_ decreased with decreasing O/C and H/C ratios (Fig. 7), and the amorphous CM samples (lignite and bituminous coal) had higher values of *μ*_p_ (~0.5) than the more crystalline anthracite and graphite samples (0.3–0.4 and 0.1–0.2, respectively). Furthermore, microstructural observations after the friction experiments revealed an absence of localized slip zones and shear fabrics (Supplementary Fig. S4), indicating that the maturity and crystallinity of individual CM grains are the major contributors to changes in friction, rather than microstructure. Thus, the relationship between the crystallinity of CM and its elemental composition is an essential controlling factor for frictional strength.

TEM observations and spectroscopic analyses of CM samples before and after friction experiments provided important insights into shear-induced CM reactions. For low-grade CM samples (lignite and bituminous coal), both the D and G bands in Raman spectra showed distinct increases in intensity after the friction experiments (Fig. 5b). These changes are similar to those caused by diagenetic maturation of low-grade CM^9^ and are consistent with previous heating experiments^52^. In contrast, TEM observations showed no significant difference in the crystallinity of low-grade CM after the friction experiments (Figs 2, 4), strongly indicating that the above changes in the Raman spectra can be attributed to carbonization during frictional slip without graphitization (crystallization)^54^. After the friction experiments at 2.0 MPa, anthracite (intermediate-grade CM) showed a dramatic increase in *I*_D_/*I*_G_ ratio (Fig. 6); this effect is also characteristic of diagenetic transformation of anthracite^9^. These reactions may be a result of shear-induced graphitization^24,27^ where the transformation of low- and intermediate-grade CM to stacks of graphene sheets is enhanced by mechanical and thermal effects during coseismic slip, thus increasing CM maturity and crystallinity. This possibility is strongly supported by the TEM observations, which show a stronger diffraction pattern and thus more strongly crystallized structures in anthracite after friction experiments at 2.0 MPa (Fig. 4o) than was the case at 0.5, 1.0 and 1.5 MPa (Figs 2c, 4c,g,k). Although graphite (high-grade CM) also showed a considerable increase in *I*_D_/*I*_G_ ratio after experiments at 1.5 and 2.0 MPa, previous friction experiments on graphite^55^ have reported similar changes in Raman spectra that have been attributed to structurally disordered graphite. Furthermore, TEM observations clearly showed that graphite after experiments at 1.5 and 2.0 MPa had a weaker diffraction pattern and thus less strongly crystallized structures (Fig. 4l,p) than graphite before and after experiments at 0.5 and 1.0 MPa (Figs 2d, 4d,h). We thus infer that the changes we observed in Raman spectra and TEM observations of graphite can be attributed to shear-induced amorphization^25,55^ and likely result in decreases of maturity and crystallinity. We therefore suggest that frictional slip at seismic slip rates increased the maturity of low- and intermediate-grade CM but decreased the maturity of high-grade CM.

Previous studies have reported that faults become weak when they contain ≥20 vol.% CM in bulk samples^31,32^. Thus, although the typical concentrations of CM in and around plate-subduction zones (≥1 vol.%; Fig. 1) might be insufficient to cause quasi-static fault weakening, the segregation of weak material into layers on the fault plane can weaken faults at much lower concentrations of CM^32,56,57^ (a few vol.%). Such layering may be a result of deposition of CM from C-H-O-rich fluids along faults^58^, diffusive mass transfer processes^14^ and formation of CM from carbonate minerals by frictional slip^36,59,60^. By considering these facts, small amounts of CM in faults are likely to dominate frictional strength of rocks in and around subduction zones in the case if CM is sufficiently enriched or segregated to form a thin layer.

**Figure 8:**
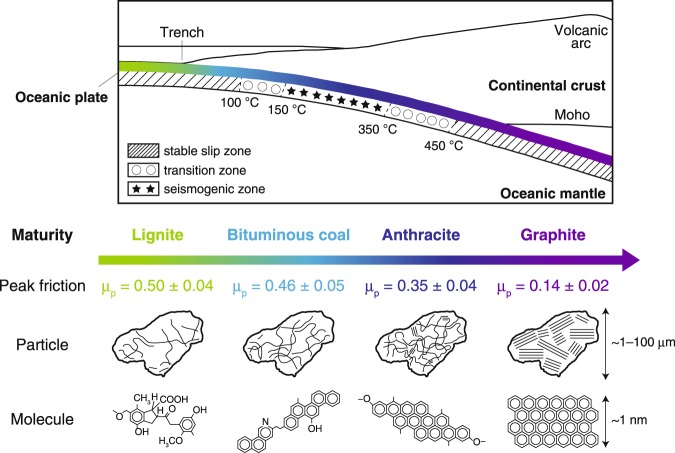
Schematic model of diagenetic transformation of CM along a subducting plate interface. Simplified tectonic cross section showing depth- and temperature-dependent changes in typical slip behaviours^77^ and progressive maturation of CM. Changes with depth of peak friction, crystallinity and representative molecular structure for each type of CM are also shown (as inferred from friction experiments, TEM observations and XRD measurements, and Matthews & Chaffee^78^, respectively).

The progress of diagenetic reactions depends mainly on temperature and burial depth. For example, the smectite to illite transformation at temperatures of 60–140 °C (ref.^61^) can lead to an increase in the frictional strength of fault rocks. The maturity and crystallinity of CM can increase with increasing temperature^9^, leading to a dramatic decrease in peak friction (Fig. 7). By considering our results together with the previous studies, we developed a schematic cross section illustrating the diagenetic transitions with increasing depth of peak frictional strength, crystallinity and representative molecular structures of CM in a subducting oceanic plate (Fig. 8). In addition, shear-induced maturation (carbonization and graphitization) of low- and intermediate-grade CM can raise the lower limit of existence areas of lignite, bituminous coal and anthracite, whereas shear-induced amorphization of high-grade CM can lower the upper limit of existence area of graphite.

The frictional data obtained from high-velocity friction experiments provide insights into the process of rupture propagation during an earthquake^37,62^. For example, fracture energy, *G*, which is calculated from the evolution of shear stress during slip, is a key factor for the control of rupture dynamics during an earthquake^63–65^. *G* is the energy required to advance a rupture front along a fault and, in the case of an “ideal” (Griffith) crack, all of the fracture energy is consumed as surface energy to generate new slip interface^66^. Some seminal studies^63,65,67^ have proposed that *G* determines rupture velocity. Theoretical calculations have suggested that the lower the ratio between *G* and total seismic energy (i.e., as *G* decreases), the higher the rupture velocity^64^, possibly because the lower peak shear strength of the fault provides less resistance to rupture propagation. To investigate the influence of CM transformation on the rupture dynamics of carbon-bearing faults, we calculated *G* for all of our experiments (with the exception of bituminous coal under 1 MPa) by using the following equation^68^:

4 $$G={\int }_{0}^{D}(\tau -{\tau }_{{\rm{d}}})d{D}^{^{\prime} }$$

The resultant values of *G* for amorphous CM samples are 1.15 ± 0.08 and 1.00 ± 0.26 MJ m^−2^ for lignite and bituminous coal, respectively, which are higher than those of partly and completely crystallized CM (0.72 ± 0.13 and 0.00 ± 0.00 MJ m^−2^ for anthracite and graphite, respectively). These results suggest that diagenetic and shear-induced maturation of CM decreases *G* and may thus increase rupture velocity in carbon-bearing faults.

The stress ratio (*S*) is another important influence on rupture propagation and is expressed as follows^10,67^:

5 $$S=({\tau }_{{\rm{y}}}-{\tau }_{{\rm{i}}})/({\tau }_{{\rm{i}}}-{\tau }_{{\rm{d}}})=({\mu }_{{\rm{y}}}-{\mu }_{{\rm{i}}})/({\mu }_{{\rm{i}}}-{\mu }_{{\rm{d}}})$$

where *τ*_y_ and *τ*_i_ are yield and initial shear stress (MPa) and *µ*_y_ and *µ*_i_ are yield and initial friction coefficient, respectively. *S* is a dimensionless parameter that controls the evolution of rupture with time and slip^67,69–71^. For example, numerical modelling of rupture propagation along a mode II crack by assuming a simple slip-weakening model and *S* of 0.8 has shown that as time and slip increase, rupture velocity passes through Rayleigh-wave velocity and finally approaches *P*-wave velocity^70^. Similarly, fault-plane modelling^69^ has suggested that a fault patch with a higher stress ratio than surrounding patches restrains rupture propagation because its higher yield shear strength or lower initial background shear strength makes rupture propagation more difficult. Thus, the stress ratio is universally applicable in studies of earthquake rupture dynamics.

We calculated stress ratios for each of the CM samples based on the experimentally determined frictional properties (Fig. 3) and Equation (5). Although high-velocity friction experiments provided only *τ*_d_, we assume that *τ*_y_ corresponds to *τ*_p_ obtained during high-velocity friction experiments. Furthermore, we can calculate average friction coefficients at low slip velocity (less than 0.056 m s^−1^) from previous experiments on pure CM, which corresponds to *µ*_i_ in Equation (5), to be 0.43 and 0.09 for amorphous and crystallized CM, respectively^27^. Therefore, we calculated the stress ratios for lignite, bituminous coal and graphite to be 0.25, 0.15 and 5.00, respectively. Importantly, this systematic increase of stress ratio with increasing maturity of CM indicates that the progressive diagenetic and shear-induced maturation of CM may inhibit rupture propagation. Thus, we can infer that higher values of *G*, lower stress ratios and lower rupture velocities are more likely to occur along the shallow part of carbon-bearing plate-boundary faults (where low-grade CM exists); these conditions are consistent with the results of analyses of dense seismic array data^72^ and two-dimensional dynamic rupture modelling^73^ of the 2011 Tohoku-Oki earthquake. Therefore, the diagenetic and shear-induced transformations of CM are important processes that may affect earthquake rupture dynamics along carbon-bearing faults.

## Conclusion

High-velocity friction experiments revealed that the peak friction of CM decreases systematically as its maturity and crystallinity increase. Diagenetic reactions of CM accompanying plate subduction can increase CM maturity and crystallinity such that carbon-bearing faults are progressively weakened with increasing depth along the subducting plate interface. Furthermore, TEM observations and spectroscopic analyses suggest that shear-induced transformation of CM can also increase maturity of low- and intermediate-grade CM whereas it can decrease maturity of high-grade CM. Experimental results also imply that the increase of maturity and crystallinity of CM in faults is likely to cause a decrease in fracture energy and an increase in stress ratio, possibly affecting the rupture process of carbon-bearing faults. We suggest that, in addition to the effect of diagenetic reactions such as the smectite to illite transformation, the diagenetic and shear-induced transformation of CM is a potential important mechanism that may affect earthquake dynamics at carbon-bearing faults such as plate-subduction boundaries. To improve our understanding of earthquake mechanics for such carbon-bearing faults, in terms of both rupture initiation and propagation, further low-velocity friction experiments on CM are needed to determine its frictional properties and its relation to the rate-and-state friction law^74,75^.

## Methods

### Extraction of CMs

Three bulk coal samples (lignite from Yasu, Japan; bituminous coal from Ashibetsu, Japan; anthracite from Ohmine, Japan) were manually powdered using an agate mortar and pestle. These samples were then treated with 18 N HCl for 1 h to remove metals, sulphides, and carbonates. The solid residues were treated with 46 N HF for 1 day to remove silicates. The precipitates were treated again with 18 N HCl for 1 day to remove fluoride by-products. Finally, the precipitates were rinsed with distilled water. After three repetitions of this rinsing process, the final solid CM residues were dried at 50 °C.

The concentrations of CM in the bulk coal samples were 90.0 ± 12.7 wt.% (lignite), 99.9 ± 2.5 wt.% (bituminous coal) and 62.8 ± 14.1 wt.% (anthracite). We used a sample of pure graphite (Wako Pure Chemical Industries product No. 072-03845) to represent fully mature CM. Finally, we sieved each of the CM powders to isolate particles of ≤75 µm size.

### Elemental analyses

Elemental compositions of the four types of CM were determined with an elemental analyser (Flash EA 1112, Thermo Finnigan). About 1 mg of dried CM wrapped in tin film was first heated to 1000 °C to dissolve the film and then combusted at 1800 °C in a quartz column. In CHN measurement mode, N_2_, CO_2_ and H_2_O gas from the heated CM were separated in a quartz tube filled with chrome oxide, reduced copper, and cobalt. In O measurement mode, a carbon nickel plate, quartz turnings, soda lime and magnesium perchlorate were used to extract CO gas from the pyrolysates in the quartz tube. Sulfanilamide (C, 41.84 wt.%; H, 4.68 wt.%; N, 16.27 wt.%; S, 18.62 wt.%; O, 18.58 wt.%) was used as a standard to obtain a calibration curve for each element. Five measurements were carried out on each type of CM to obtain mean values and standard deviations of the atomic ratios of O/C and H/C.

### Transmission electron microscope observations

The transmission electron microscope (TEM; JSM-2100, JEOL) we used was operated at an acceleration voltage of 200 kV with a magnification of 500,000. Before TEM observations, a copper microgrid was dried for ≥1 day at 40 °C under vacuum (≤10 Pa).

### X-ray diffraction analyses

X-ray diffraction (XRD) profiles of CM were obtained by using a Spectris PANalytical X’Pert PRO MPD spectrometer with monochromatised CuKα radiation operated at tube voltage of 45 kV and tube current of 40 mA. The scan range was 3°–75° (Δ2θ), an angle resolution of 0.001°, a scan rate of 0.1° s^−1^ and a step width of 0.008°. Before analysis, each CM sample was dried at 50 °C for several hours. A zero-diffraction plate (made from silicon) was used as a sample holder.

### Calculation of frictional properties

The mechanical data, after subtracting the background noise and friction between the Berea sandstone and the polytetrafluoroethylene sleeve (Supplementary Fig. S2), were fitted by the empirical exponential power law described by Equation (1) using a least-squares fitting method. Although this is a broadly accepted method for fitting curves to mechanical data obtained in high-speed friction experiments^27,31,35^, the choice of fitting variables is a critical concern. We therefore fitted the measured mechanical data for four sets of variables: (i) *τ*_p_, *τ*_d_, and *D*_c_, (ii) *τ*_d_ and *D*_c_, (iii) *τ*_p_ and *D*_c_ and (iv) *D*_c_. Fitting curves of representative mechanical data for these four sets of variables are shown in Supplementary Figure S5. For the cases where *τ*_p_ and *τ*_d_ were constant, we adopted the values of the maximum shear stress at the initial stage of the experiment and the average shear stress at displacement of 14–15 m for *τ*_p_ and *τ*_d_, respectively (Supplementary Fig. S5). The coefficient of determination (*R*^2^) is defined as follows:

6 $${R}^{2}=1-\frac{{\sum }^{}{({\tau }_{{\rm{meas}}}-{\tau }_{{\rm{calc}}})}^{2}}{{\sum }^{}{({\tau }_{{\rm{meas}}}-{\tau }_{{\rm{mean}}})}^{2}}$$

where *τ*_meas_ is the measured shear stress (MPa), *τ*_calc_ is the shear stress calculated by using a least-squares fitting method (MPa) and *τ*_mean_ is the shear stress obtained by averaging the measured shear stress (MPa). Supplementary Figure S3 shows all of the measured mechanical data for the four fitting curves for each normal stress and each type of CM. Supplementary Figure S6 shows the fitting lines and curves for the estimated *τ*_p_, *τ*_d_, and *D*_c_ values for each type of CM and the four sets of variables. Supplementary Tables S2–S5 summarise the frictional properties we obtained for lignite, bituminous coal, anthracite and graphite, respectively. Among the four fitting methods, using *τ*_p_, *τ*_d_ and *D*_c_ as variables resulted in the best fitting curve with the highest *R*^2^ value for all stress conditions. Therefore, we adopted *τ*_p_, *τ*_d_ and *D*_c_ as variables in our calculations of frictional properties.

### Microstructural observations

For microstructural observations on the polished surfaces of all types of CM samples after friction experiments under 1 MPa normal stress, we operated a SEM (JCM-6000PLUS, JEOL) with an acceleration voltage of 10 kV and magnifications of 40 and 400. Each SEM images are almost parallel to the slip direction (Supplementary Fig. S1b).

### Infrared spectroscopy

We used a Fourier transform IR spectrometer (FT/IR-4700, Jasco Inc.) equipped with an IR microscope (IRT-5200, Jasco Inc.) to obtain IR absorbance spectra of CM samples before and after our friction experiments. Before the IR absorbance measurements, the CM samples and CaF_2_ plates were dried at 50 °C for 1 day. CM samples were placed on a plate and then hand-pressed with another plate to prevent saturation. To acquire one IR spectrum, 100 spectra were accumulated each with exposure time of 1 s, wavenumber resolution of 4 cm^−1^, wavenumber range of 4000–1000 cm^−1^, and aperture size of 50 × 50 µm^2^. Background intensities of the IR spectra were eliminated by measuring a blank CaF_2_ plate.

### Raman spectroscopy

We used a Raman microspectrometer (XploRA, Horiba Jobin Yvon Inc.) equipped with a laser (532 nm) to obtain Raman spectra of CM samples before and after the friction experiments. Before the Raman spectra measurements, samples were dried at 50 °C for 1 day. To acquire one Raman spectrum, 10 spectra were accumulated with an exposure time of 10 s and a laser power of 0.10 mW to avoid thermal damage on the targeted surface of CM samples. We obtained Raman spectra at 30 points per sample. We followed the M-1 Method of Henry *et al*.^76^ (with the exception of the background correction function) to determine the Raman spectral parameters. Henry *et al*.^76^ reported that the peak deconvolution method can yield unnecessary errors that are derived from operator manipulation, especially for analysis of low-grade CM. We used PeakFit 3.0 software (Systat Software Inc.) for smoothing of spectra and a linear baseline correction of 1000–1800 cm^−1^ (Supplementary Fig. S7). We determined values of *I*_D_ and *I*_G_ as the maximum intensities of a corrected spectrum at 1325–1375 cm^−1^ and 1575–1625 cm^−1^, respectively, and then calculated *I*_D_/*I*_G_ ratios (*n* = 30).

## Data Availability

The data that support the findings of this study are available in the main text, figures, tables and references or on request from the corresponding author.

## Supplementary information

Dataset 1
