# Supplementary material for: Diagenetic and shear-induced transitions of frictional strength of carbon-bearing faults and their implications for earthquake rupture dynamics in subduction zones
Source: Sci Rep. 2019 May 27;9:7884. doi: 10.1038/s41598-019-44307-y (PMC6536680; doi:10.1038/s41598-019-44307-y)
Supplement: Supplementary file 1 — Dataset 1 [file 41598_2019_44307_MOESM1_ESM.pdf]

Supplementary Information

Diagenetic and shear-induced transitions of frictional strength of carbon-bearing faults and their implications for earthquake rupture dynamics in subduction zones

Shunya Kaneki[1,2*] and Tetsuro Hirono[1]

[1] Department of Earth and Space Science, Graduate School of Science, Osaka University, Toyonaka, Osaka 560-0043, Japan

[2] Present address: Disaster Prevention Research Institute, Kyoto University, Uji, Kyoto 611-0011, Japan

* E-mail: kaneki.shunya.62a@st.kyoto-u.ac.jp

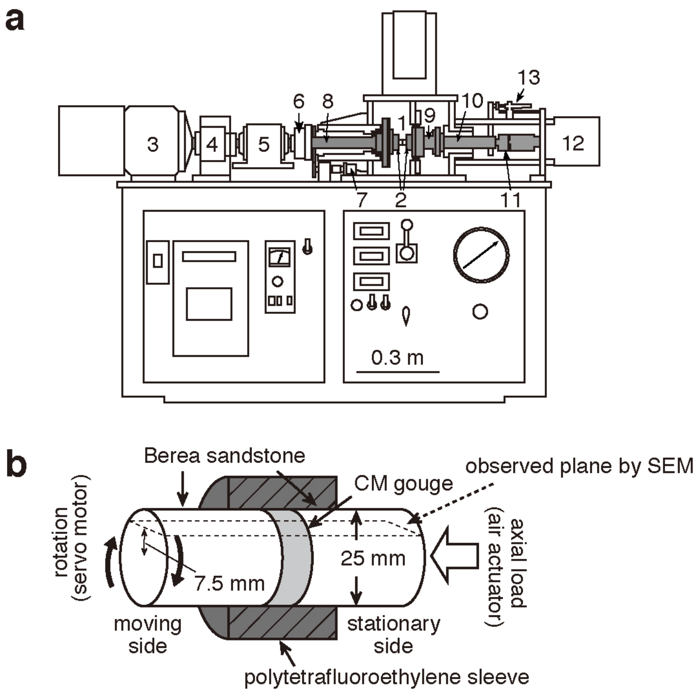

Supplementary Figure S1. Friction apparatus and sample assemblage. (**a**) Simplified sketch of high-velocity rotary friction apparatus at Yamaguchi University (modified from Mizoguchi et al.[79]). 1, sample; 2, sample holder; 3, motor; 4, torque limiter; 5, torque gauge; 6, electromagnetic clutch; 7, rotary encoder; 8, rotary column; 9, torque-axial force gauge; 10, ball spin; 11, axial force gauge; 12, air actuator; 13, displacement transducer. (**b**) Detailed view of sample assemblage.

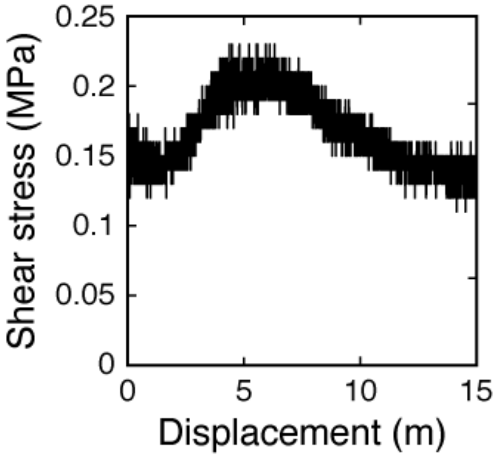

Supplementary Figure S2. Change of shear stress of polytetrafluoroethylene sleeve with slip. Mechanical data used for correction of torque between the polytetrafluoroethylene sleeve and the Berea sandstone (Supplementary Fig. S1b).

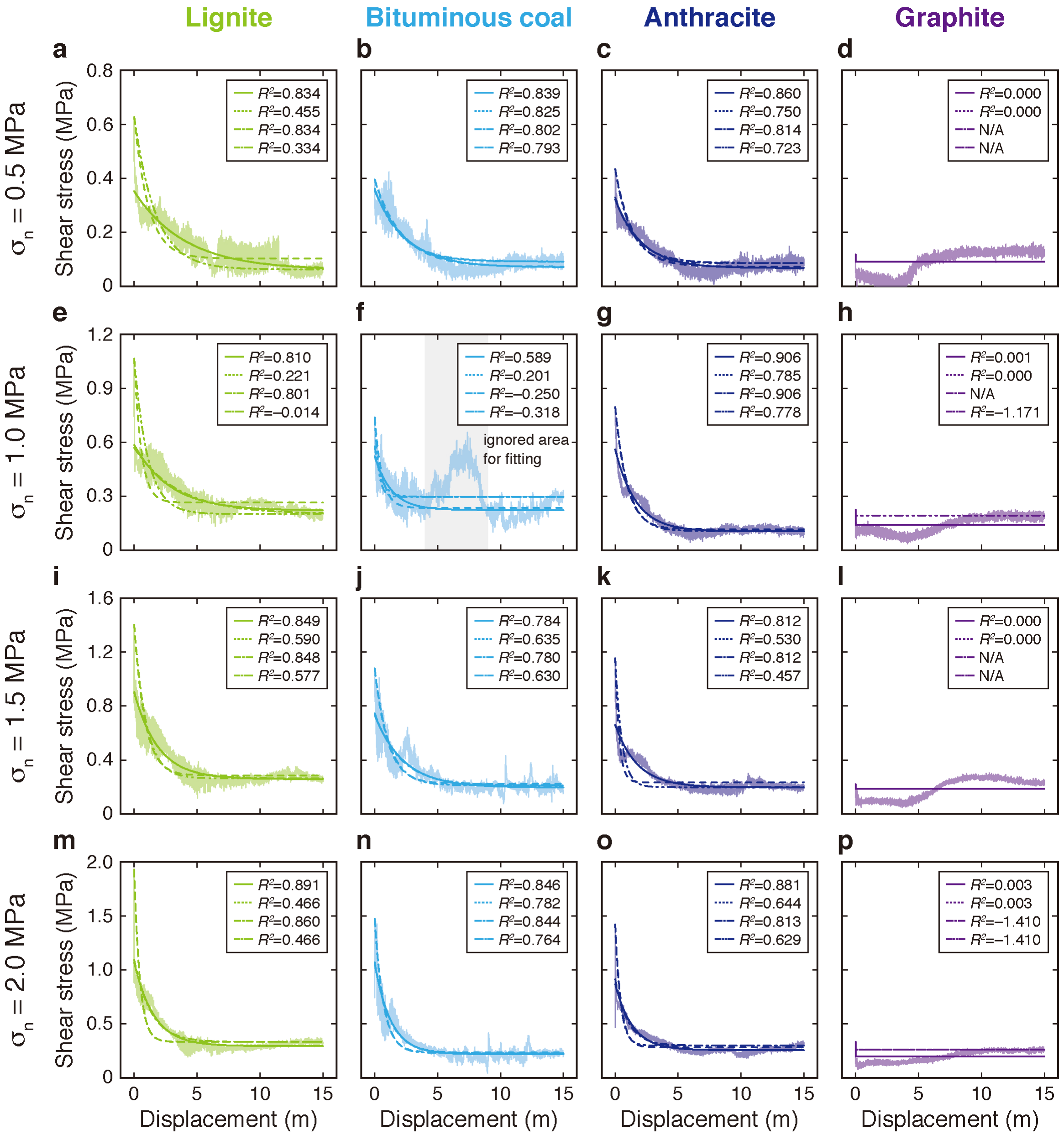

Supplementary Figure S3. Results of curve fittings for all experiments. Normal stresses applied were (**a–d**) 0.5 MPa, (**e–h**) 1.0 MPa, (**i–l**) 1.5 MPa, and (**m–p**) 2.0 MPa. Each graph shows fitting curves derived by applying equation (1) with four different sets of variables. Coefficients of determination (R^2 values) were calculated using equation (6) in Methods.

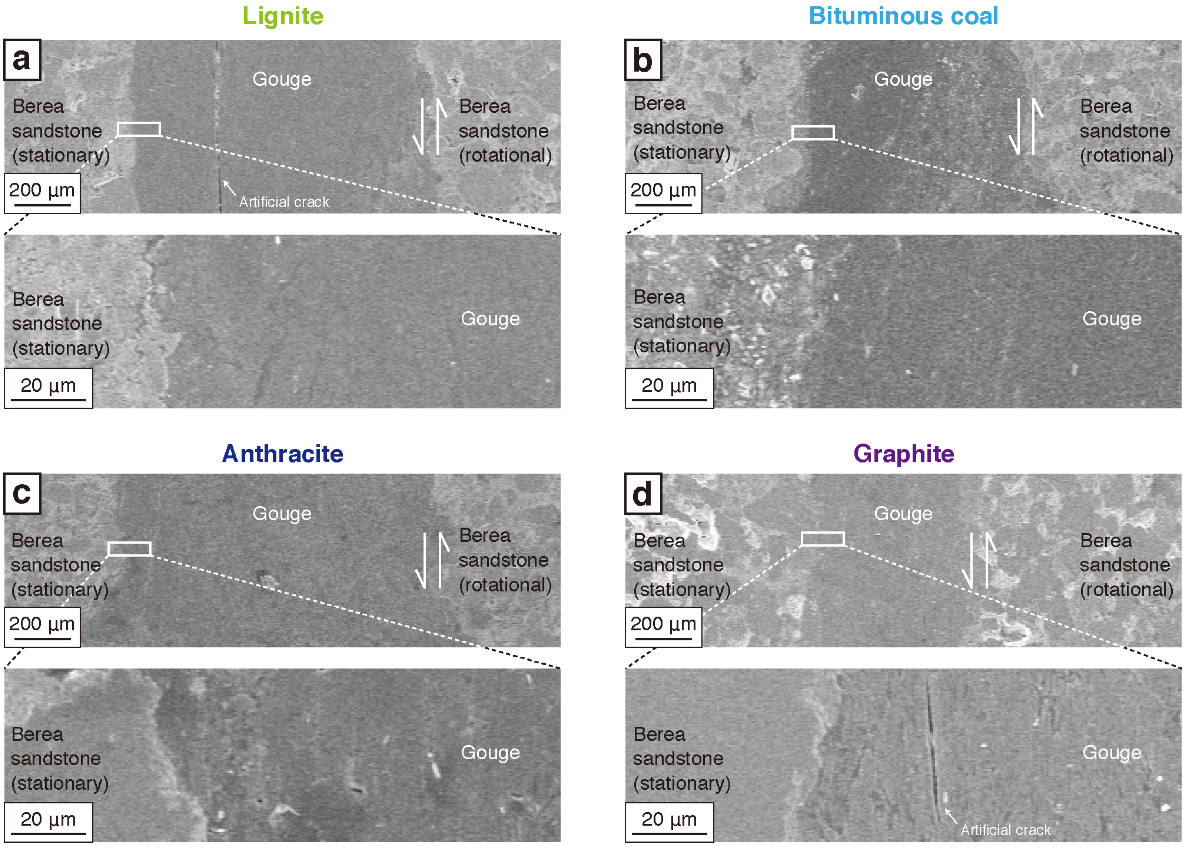

Supplementary Figure S4. Microstructures of CM samples after friction experiments. Results of SEM observations of the polished surfaces of (**a**) lignite, (**b**) bituminous coal, (**c**) anthracite and (**d**) graphite after experiments at normal stress of 1.0 MPa. In each case, the CM in the slip zone has a homogeneous elemental distribution and is optically opaque, so it is unlikely that use of either optical microscope or backscattered electron imaging under an electron microscope would be useful. Cracks in the lignite and graphite samples were probably caused by the release of axial normal stress after the experiments. Neither shear fabrics (such as R1 or Y shear planes) nor intense shear localization was observed. The slip zones were 0.7–1.0 mm thick.

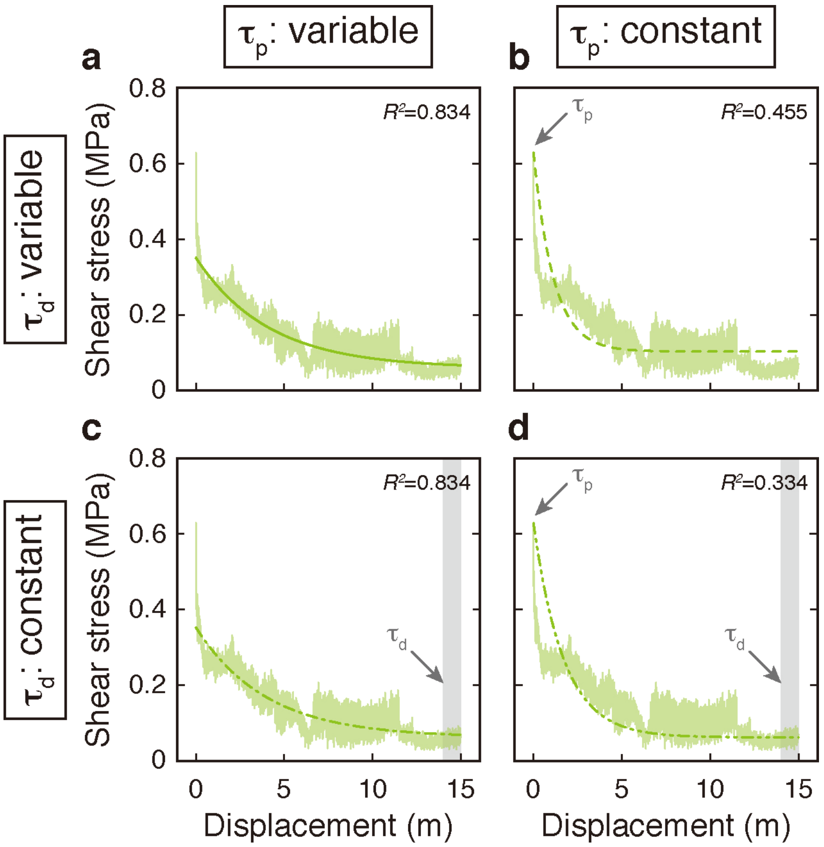

Supplementary Figure S5. Four patterns for fitting of data from friction experiments. Variables for fitting were (**a**) τ_p, τ_d, and D_c, (**b**) τ_d and D_c, (**c**) τ_p and D_c, and (**d**) D_c. Data of shear stress with slip shown are from lignite at 0.5 MPa normal stress. Coefficients of determination (R^2 values) are calculated by using equation (6) in Methods.

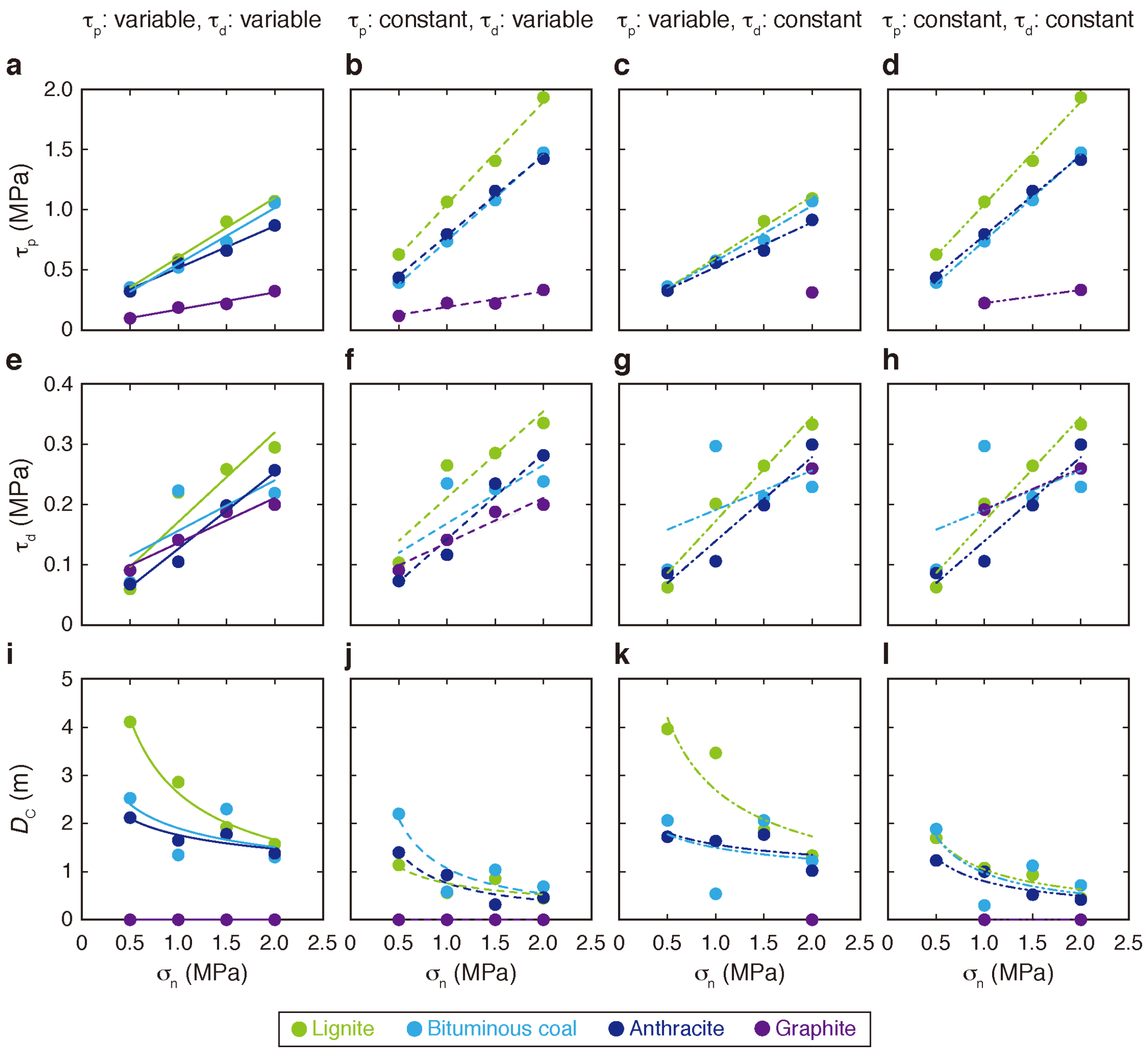

Supplementary Figure S6. Determination of frictional properties for variable fitting conditions and CM types. Linear correlations of normal stress with (**a–d**) peak shear stress and (**e–h**) dynamic shear stress were obtained using equation (2). Exponential correlation of normal stress with critical slip-weakening distance (**i–l**) were obtained using equation (3). All values of the frictional properties presented are summarized in Supplementary Tables S2–S5.

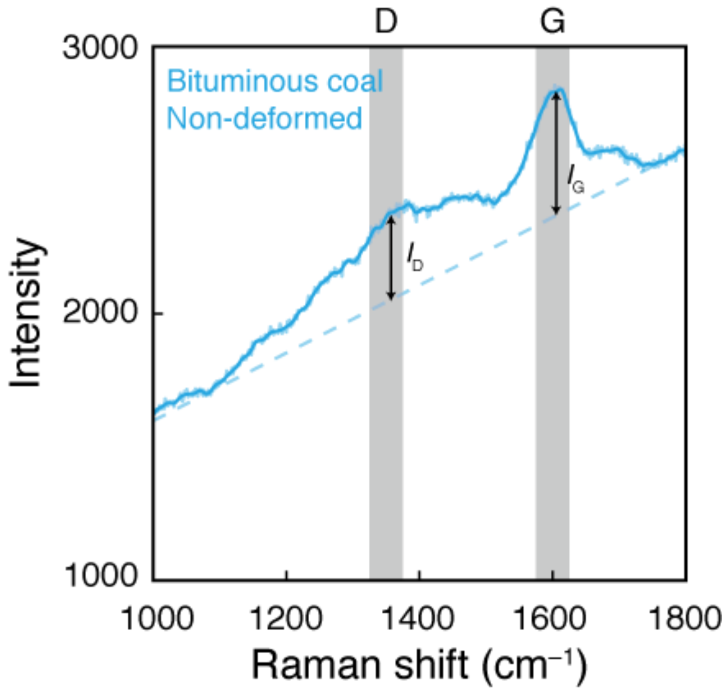

Supplementary Figure S7. Methodology of calculating I_D/I_G ratio. Obtained Raman spectrum (transparent light-blue line) was smoothed by using the FFT Filtering method with smoothing degree of 25 % (opaque light-blue line). After correcting background with a linear function (dashed light-blue line; in this case, *Background intensity* = 1.2696 × *Raman shift* – 329.4450), the peak intensities of D and G bands, I_D and I_G, were determined as the maximum intensities of the corrected spectrum at 1325–1375 cm^{-1} and 1575–1625 cm^{-1}, respectively.

Supplementary Table S1. Summary of elemental compositions of the CM samples.

Sample	C (mol kg^{-1})	H (mol kg^{-1})	O (mol kg^{-1})	O/C	H/C
Lignite	48.59	94.00	16.76	0.37 ± 0.03	1.93 ± 0.11
	46.21	95.33	18.37		
	44.72	88.88	17.76		
	48.31	85.11	16.81		
	49.71	94.45	18.32		
Bituminous coal	68.77	93.49	4.66	0.07 ± 0.00	1.58 ± 0.16
	65.48	100.17	4.56		
	68.51	114.42	4.43		
	69.05	108.50	4.64		
	68.94	122.25	4.61		
Anthracite	73.41	53.01	1.91	0.03 ± 0.00	0.74 ± 0.08
	72.83	50.59	1.79		
	73.26	63.28	1.95		
	73.40	47.11	1.84		
	73.30	56.97	1.95		
Graphite	83.37	0.00	0.00	0.00 ± 0.00	0.00 ± 0.00
	83.70	0.00	0.00		
	83.20	0.00	0.00		
	83.42	0.00	0.00		
	83.53	0.00	0.00		

Supplementary Table S2. Summary of frictional properties determined for lignite for all curve-fitting cases with different sets of variables and normal stresses.

Sample	Variables	σ_n (MPa)	τ_p (MPa)	τ_d (MPa)	D_c (m)	μ_p	μ_d	α	β
Lignite	τ_p, τ_d, D_c	0.5	0.351	0.060	4.103	0.50 ± 0.04	0.15 ± 0.04	2.63	0.66
		1.0	0.586	0.220	2.855				
		1.5	0.901	0.258	1.918				
		2.0	1.072	0.295	1.566				
	τ_d, D_c	0.5	0.629	0.103	1.136	0.85 ± 0.05	0.14 ± 0.04	0.76	0.53
		1.0	1.066	0.265	0.579				
		1.5	1.405	0.285	1.036				
		2.0	1.932	0.335	0.689				
	τ_p, D_c	0.5	0.353	0.063	3.963	0.51 ± 0.04	0.17 ± 0.03	2.69	0.64
		1.0	0.569	0.201	3.465				
		1.5	0.905	0.264	1.861				
		2.0	1.094	0.333	1.333				
	D_c	0.5	0.629	0.063	1.698	0.85 ± 0.05	0.17 ± 0.03	1.04	0.73
		1.0	1.066	0.201	1.075				
		1.5	1.405	0.264	0.934				
		2.0	1.932	0.333	0.715				

Supplementary Table S3. Summary of frictional properties determined for bituminous coal for all curve-fitting cases with different sets of variables and normal stresses.

Sample	Variables	σ_n (MPa)	τ_p (MPa)	τ_d (MPa)	D_c (m)	μ_p	μ_d	α	β
Bituminous coal	τ_p, τ_d, D_c	0.5	0.356	0.071	2.522	0.46 ± 0.05	0.08 ± 0.05	1.90	0.33
		1.0	0.522	0.223	1.344				
		1.5	0.733	0.197	2.297				
		2.0	1.057	0.219	1.300				
	τ_d, D_c	0.5	0.396	0.073	2.197	0.72 ± 0.02	0.10 ± 0.06	1.07	0.98
		1.0	0.739	0.235	0.579				
		1.5	1.079	0.225	1.036				
		2.0	1.474	0.238	0.689				
	τ_p, D_c	0.5	0.362	0.092	2.064	0.46 ± 0.05	0.07 ± 0.08	1.50	0.25
		1.0	0.577	0.297	0.539				
		1.5	0.746	0.212	2.064				
		2.0	1.071	0.229	1.225				
	D_c	0.5	0.396	0.092	1.884	0.72 ± 0.02	0.07 ± 0.08	0.97	0.83
		1.0	0.739	0.297	0.297				
		1.5	1.079	0.212	1.122				
		2.0	1.474	0.229	0.715				

Supplementary Table S4. Summary of frictional properties determined for anthracite for all curve-fitting cases with different sets of variables and normal stresses.

Sample	Variables	σ_n (MPa)	τ_p (MPa)	τ_d (MPa)	D_c (m)	μ_p	μ_d	α	β
Anthracite	τ_p, τ_d, D_c	0.5	0.322	0.068	2.118	0.35 ± 0.04	0.13 ± 0.02	1.76	0.26
		1.0	0.560	0.105	1.646				
		1.5	0.661	0.198	1.775				
		2.0	0.870	0.257	1.378				
	τ_d, D_c	0.5	0.435	0.073	1.395	0.66 ± 0.03	0.14 ± 0.02	0.76	0.91
		1.0	0.797	0.116	0.931				
		1.5	1.156	0.235	0.315				
		2.0	1.423	0.282	0.462				
	τ_p, D_c	0.5	0.329	0.086	1.724	0.37 ± 0.04	0.14 ± 0.03	1.56	0.21
		1.0	0.561	0.106	1.634				
		1.5	0.661	0.199	1.769				
		2.0	0.916	0.299	1.020				
	D_c	0.5	0.435	0.086	1.232	0.66 ± 0.03	0.14 ± 0.03	0.80	0.69
		1.0	0.797	0.106	0.997				
		1.5	1.156	0.199	0.521				
		2.0	1.423	0.299	0.419				

Supplementary Table S5. Summary of frictional properties determined for graphite for all curve-fitting cases with different sets of variables and normal stresses.

Sample	Variables	σ_n (MPa)	τ_p (MPa)	τ_d (MPa)	D_c (m)	μ_p	μ_d	α	β
Graphite	τ_p, τ_d, D_c	0.5	0.099	0.091	0.000	0.14 ± 0.02	0.08 ± 0.01	0.000	0.000
		1.0	0.188	0.141	0.000				
		1.5	0.219	0.188	0.000				
		2.0	0.325	0.200	0.000				
	τ_d, D_c	0.5	0.119	0.091	0.000	0.13 ± 0.03	0.08 ± 0.01	0.000	0.000
		1.0	0.226	0.141	0.000				
		1.5	0.222	0.188	0.000				
		2.0	0.334	0.200	0.000				
	τ_p, D_c	0.5	N/A	N/A	N/A	N/A	N/A	N/A	N/A
		1.0	N/A	N/A	N/A				
		1.5	N/A	N/A	N/A				
		2.0	0.314	0.260	0.000				
	D_c	0.5	N/A	N/A	N/A	0.11	0.07	0.000	0.000
		1.0	0.226	0.192	0.000				
		1.5	N/A	N/A	N/A				
		2.0	0.334	0.260	0.000				

Supplementary references

79. Mizoguchi, K., Hirose, T., Shimamoto, T. & Fukuyama E. High-velocity frictional behavior and microstructure evolution of fault gouge obtained from Nojima fault, southwest Japan. *Tectonophysics* **471**, 285–296 (2009).
